# Optimizing the early detection of low pathogenic avian influenza H7N9 virus in live bird markets

**DOI:** 10.1098/rsif.2021.0074

**Published:** 2021-05-05

**Authors:** Claire Guinat, Damian Tago, Tifenn Corre, Christian Selinger, Ramsès Djidjou-Demasse, Mathilde Paul, Didier Raboisson, Thuy Nguyen Thi Thanh, Ken Inui, Long Pham Thanh, Pawin Padungtod, Timothée Vergne

**Affiliations:** ^1^ IHAP, Université de Toulouse, INRAE, ENVT, Toulouse, France; ^2^ FAO, Bangkok, Thailand; ^3^ US-ODR, INRAE, Castanet Tolosan, France; ^4^ MIVEGEC, Université Montpellier, IRD, CNRS, Montpellier, France; ^5^ FAO, Country Office for Viet Nam, Hanoi, Vietnam; ^6^ FAO, Department of Animal Health (DAH), Ministry of Agriculture and Rural Development (MARD), Hanoi, Vietnam; ^7^ DAH, MARD, Hanoi, Vietnam

**Keywords:** transmission model, low pathogenic H7N9 avian influenza virus, surveillance strategies, live-bird markets

## Abstract

In Southeast Asia, surveillance at live bird markets (LBMs) has been identified as crucial for detecting avian influenza viruses (AIV) and reducing the risk of human infections. However, the design of effective surveillance systems in LBMs remains complex given the rapid turn-over of poultry. We developed a deterministic transmission model to provide guidance for optimizing AIV surveillance efforts. The model was calibrated to fit one of the largest LBMs in northern Vietnam at high risk of low pathogenic H7N9 virus introduction from China to identify the surveillance strategy that optimizes H7N9 detection. Results show that (i) using a portable diagnostic device would slightly reduce the number of infected birds leaving the LBM before the first detection, as compared to a laboratory-based diagnostic strategy, (ii) H7N9 detection could become more timely by sampling birds staying overnight, just before new susceptible birds are introduced at the beginning of a working day, and (iii) banning birds staying overnight would represent an effective intervention to reduce the risk of H7N9 spread but would decrease the likelihood of virus detection if introduced. These strategies should receive high priority in Vietnam and other Asian countries at risk of H7N9 introduction.

## 1. Introduction

New avian influenza A virus (AIV) strains continue to pose serious clinical and economic challenges to global public and animal health, and leading to substantial economic losses. Since 2013, a novel avian-origin H7N9 virus has emerged in eastern China, causing severe respiratory disease and fatalities in humans [1,2]. China experienced five epidemic waves from 2013 to 2017, during which the number of reported cases has increased significantly, reaching over 1600 human infections [3,4]. During the first four waves, the H7N9 AIV circulating in Chinese poultry were low-pathogenic (LP) with asymptomatic infection in poultry [4,5]. During the fifth wave in 2017, a highly pathogenic (HP) H7N9 AIV variant emerged [6–8]. Such rapid increase in the number of H7N9 human infections and the emergence of H7N9 as HP AIV variant have raised fear of a pandemic threat [9].

In Asia, live bird markets (LBMs) are known as high-risk places for the transmission, evolution and maintenance of AIV [10,11]. In China, most human H7N9 cases have been associated with previous exposure to poultry at LBMs [12–14]. In Chinese LBMs, H7N9 virus has been extensively detected in chickens [15,16], which are the primary source of H7N9 infection in humans [17,18]. Frequent interactions among different poultry species and humans at LBMs provide an ideal interface for transmission of AIV and emergence of new variants by mixing AIV from different sources [12,16,19].

Systematic surveillance at LBMs remains essential for detecting novel AIV and reducing the risk of human infections [16,20,21]. Optimizing AIV surveillance strategies in LBMs has mainly focused so far on identifying the most sensitive sample materials [11,22] and on increasing diagnostic assay sensitivity [23]. However, lack of knowledge remains on how the sampling design (i.e. sampling time and sample size) could be optimized to maximize the probability to detect AIV, given the rapid turn-over of poultry populations in LBMs. Moreover, the cost of surveillance, which is a key parameter for policy decision and surveillance design, should be taken into consideration when assessing different surveillance strategies, especially in low- and middle-income settings.

Given the sharp increase in the number of H7N9 outbreaks in China in 2013–2017 and regular cross-border trade of birds originating from China [24], the rapid detection of emerging AIV was crucial to minimize public health and economic impact in Vietnam. The current surveillance programme for H7N9 in Vietnam involved biweekly random sampling of chickens in LBMs with value chain linkages to China. All samples were transported to an official diagnostic laboratory where they were consecutively screened for M, H7 and N9 genes using RT-PCR. As an alternative to this surveillance strategy, a portable PCR device has been introduced recently to improve H7N9 detection and response capacities in Vietnam [25,26]. This device can be directly deployed in LBMs and allows virus detection within 7 h after sampling, while the laboratory-based surveillance protocol takes on average 72 h due to logistic reasons.

In the light of the introduction of this innovative diagnostic tool, the general objective of this study was to compare the weekly costs and effectiveness of different surveillance strategies at LBMs, with the view to identify the strategy that optimizes H7N9 detection. To do this, a deterministic transmission model of AIV including environmental shedding and bird trading was developed and calibrated to fit one of the largest LBMs in northern Vietnam at high risk of LP H7N9 introduction from China.

## 2. Material and methods

### 2.1. Structure of the within-LBM H7N9 transmission model

#### 2.1.1. Dynamics of bird trading in the LBM

**Figure 1.**
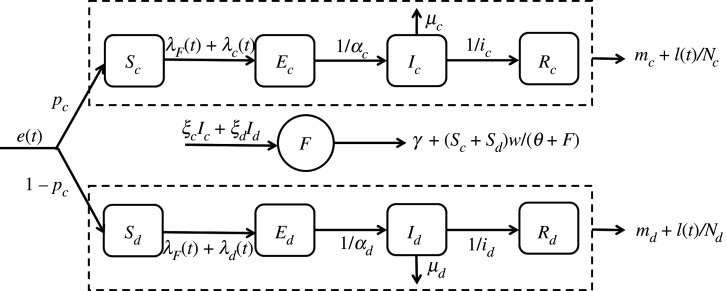
Flux diagram of the full dynamic transmission model. Parameters involved in the formulation of transition rates between compartments are: *e*(*t*) being the entry rate, *p_c_* (resp. 1 − *p_c_*) being the proportion of chickens (resp. ducks), *λ_F_* being the force of infection due to environmental contamination, *λ_c_* (resp. *λ_d_*) being the force of infection due to contacts with infectious chickens (resp. ducks), *α_c_* (resp. *α_d_*) being the average duration of the latent period in chickens (resp. ducks), *µ_c_* (resp. *µ_d_*) being the mortality rate due to H7N9 infection in chickens (resp. ducks), *i_c_* (resp. *i_d_*) being the average duration of the infectious period in chickens (resp. ducks), *m_c_* (resp. *m_d_*) being the natural mortality rate for chickens (resp. ducks), *l*(*t*) being the exit rate, *N_c_* (resp. *N_d_*) being the total number of chickens (resp. ducks), *ξ_c_* (resp. *ξ_d_*) being the excretion rate of infectious doses by chickens (resp. ducks), *I_c_* (resp. *I_d_*) being the number of infectious chickens (resp. ducks), *γ* being the virus inactivation rate, *S_c_* (resp. *S_d_*) being the number of susceptible chickens (resp. ducks), *w* being the contact rate with one infectious dose in the environment, *θ* being the number of infectious doses that are necessary to infect a bird and *F* being the number of infectious doses present in the environment.

It was assumed that chickens and ducks were introduced into the LBM at an entry rate *e*(*t*) that depended on the time of the day, and moved out for slaughter and trade at a time-varying exit rate *l*(*t*) (figure 1; electronic supplementary material, appendix)*.* The model formulation also allowed a proportion of the bird populations to stay overnight in the LBM and therefore to contribute potentially to the maintenance and amplification of the virus. Among the birds leaving the market, it was considered that a given proportion was traded to farms and other LBMs. These birds were assumed to be those representing the highest risk for secondary spread outside the studied LBM.

#### 2.1.2. Dynamics of H7N9 transmission in the LBM

Once the virus was introduced into an LBM, chickens and ducks were assumed to pass through four infection stages: susceptible (*S*_*c*_ and *S*_*d*_), infected but not yet infectious (*E*_*c*_ and *E*_*d*_), infectious (*I*_*c*_ and *I*_*d*_) and recovered (*R*_*c*_ and *R*_*d*_). All birds that were brought into the LBM were assumed to be susceptible. Susceptible birds (S) could become infected (E) during an average latent period duration (*α_d_* and *α_c_*) after which they were infectious (I) during an average infectious period duration (*i_d_* and *i_c_*). Subsequently, they could die due to H7N9 infection (with a rate *µ_c_* and *µ_d_*) or recovered (R) from the infection. Note that all birds (whatever the compartment S, E, I or R) could leave the LBM for slaughter and trade at time-varying exit rates *l*(*t*) or could die from natural causes other than H7N9 infection (with a rate *m_c_* and *m_d_*). Viral persistence of H7N9 in the environment has been recently reported [27] and several studies have demonstrated the importance of environmental transmission as a driver of AIV outbreaks [28–30]. Therefore, it was assumed that infectious ducks and chickens could contaminate the environment (F) by excreting infectious doses at an excretion rate *ξ_d_* and *ξ_c_*, respectively, with *ξ_d_* < *ξ_c_*, since ducks were reported excreting less H7N9 virus than chickens [24]. The amount of infectious doses in the environment was assumed to decrease at an inactivation rate *γ*.

The force of infection, determining the rate at which susceptible birds moved to the infected compartment (E), was defined as the sum of the forces of infection due to environmental contamination (indirect transmission) and due to contacts with infectious birds (direct transmission) [31]. Firstly, the force of infection due to environmental contamination (*λ_F_*) was adapted from [32,33] using Hill-type function:

$$\lambda_{F}=\frac{w\;*\;F}{\theta +F},$$

with *w* being the contact rate with one infectious dose in the environment, *θ* being the number of infectious doses that are necessary to infect a bird and *F* being the number of infectious doses present in the environment. Note that an infection via the contaminated environment could occur in the absence of infectious birds. Secondly, the force of infection due to contacts with infectious chickens (*λ_c_*) and ducks (*λ_d_*) was given by

$$\lambda_{j}=\beta_{j}\frac{I_{c}+I_{d}}{N_{c}+N_{d}};\;j\in \{c,\;d\},$$

with *β_j_* being the infection rate for chickens (*j* = *c*) and ducks (*j* = *d*).

The full dynamic transmission model is defined by the flux diagram in figure 1 (see electronic supplementary material, appendix, for the full mathematical specification of the model).

### 2.2. Calibration of the within-LBM H7N9 transmission model

#### 2.2.1. Parameters for the bird trading process

**Figure 2.**
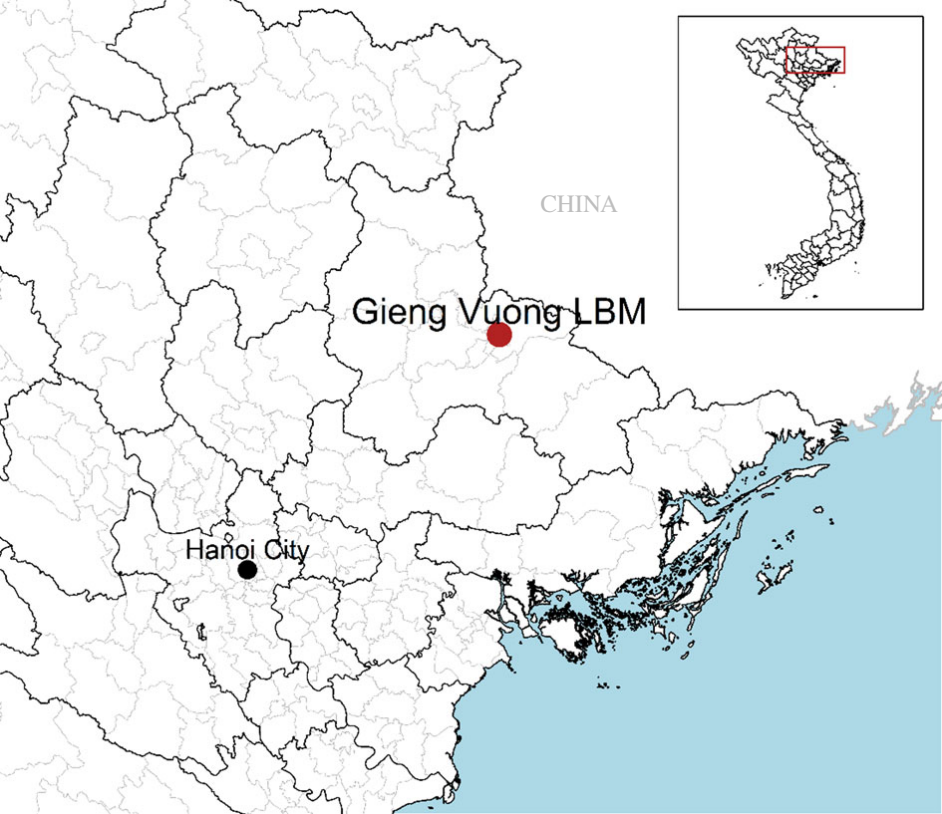
Geographical location of Giếng Vuông LBM, Lang Son province, northern Vietnam.

The parameters related to the population dynamics of birds were derived from field observations conducted at Giếng Vuông LBM, one of the largest LBMs in Lang Son province in northern Vietnam (figure 2). This LBM was identified by Vietnamese veterinary services as at high risk of H7N9 introduction due to potential cross-border trade of birds from China [24], and thus represented a relevant candidate for this study. A questionnaire survey was developed to collect information from LBM managers and traders on population dynamics of birds. Interviews were conducted in Vietnamese language by a native Vietnamese speaker proficient in English seconded by the first author and were facilitated by representatives from the District Veterinary Station (DVS) and the Sub-Department of Animal Health (SDAH). The LBM manager and staff were interviewed together, with answers agreed upon consensus while eight poultry traders were randomly selected and individually interviewed. Direct observations were made during the visits and used to cross-check interviewees' answers. Due to the nature of the study and the low risk posed to the participants, formal approval from an ethics committee was not a requirement at the time of the study. The questionnaire is available upon request to the corresponding author.

#### 2.2.2. Parameters for the H7N9 transmission process

Most of the parameters related to H7N9 transmission were derived from the published literature. Values and references are summarized in table 1 and details can be found in electronic supplementary material, appendix. Based on the transmission model, the basic reproduction number (*R*_0_), defined as the average number of secondary infections caused by a single infectious individual in a fully susceptible population [31], was formulated as a function of all model parameters using a next-generation matrix approach [40]. Assuming that the *R*_0_ had been estimated around 4.1 for H7N9 bird-to-bird transmission in LBMs [34], the lower bound value for *w* was thus estimated at 10^−4^ (electronic supplementary material, appendix). The sensitivity analysis was performed for parameters for which limited information was available from the published literature (i.e. *Q*, *ξ* and *w*) and presented in detail in electronic supplementary material, appendix.

**Table 1.** Parameter values related to H7N9 transmission model.

parameter	description	value (unit)	subtype	reference
d*t*	step-time	hour		
*m_c_*	natural mortality rate for chickens attributable to other causes than H7N9 infection (per hour)	10^−4^	—	—
*m_d_*	natural mortality rate for ducks attributable to other causes than H7N9 infection (per hour)	10^−4^	—	—
*β_c_*	infection rate for chickens (per hour)	0.02	H7N9	[34]
*β_d_*	infection rate for ducks (per hour)	*β_c_* . *K*	H7N9	[24,27]
*K*	ratio of infection rate for ducks versus chickens	0.8	H7N9	[24,27]
*α_c_*	average duration of the latent period for chickens (hours)	14.9	H5N1	[35]
*α_d_*	average duration of the latent period for ducks (hours)	14.9	H5N1	[35]
*µ_c_*	mortality rate for chickens due to infection (per hour)	10^−4^	H7N9	[27,36,37]
*µ_d_*	mortality rate for ducks due to infection (per hour)	10^−4^	H7N9	[27]
*i_c_*	average duration of the infectious period for chickens (hours)	192	H7N9	[24]
*i_d_*	average duration of the infectious period for ducks (hours)	120	H7N9	[24]
*γ*	inactivation rate (per hour)	0.01	AIV	[38,39]
*ξ_c_*	number of infectious doses excreted by chickens (per hour)	1	—	—^a^
*ξ_d_*	number of infectious doses excreted by ducks (per hour)	*ξ_c_* . *Q*	—	—^a^
*Q*	ratio of excretion rate for ducks versus chickens	0.8	H7N9	[24,27]^a^
*w*	contact rate with one infectious dose (per hour)	10^−4^	—	—^a^
*θ*	number of infectious doses to infect chickens and ducks	1	—	—

^a^See electronic supplementary material, appendix, for sensitivity analysis regarding these parameter assumptions.

### 2.3. Simulation of the within-LBM H7N9 transmission model

The model formulation was embedded within a deterministic framework. It assumed homogeneous mixing, i.e. birds uniformly and randomly contact each other, be they ducks or chickens. The model was initialized by introducing one infectious chicken at the moment of the day with the highest entry rate in the LBM and used to simulate the number of birds in each infection stage. The model was implemented in the R programming language [41].

### 2.4. Cost-effectiveness of surveillance strategies

#### 2.4.1. Definition of the different surveillance strategies

The following surveillance strategies were incorporated into the model to assess their cost-effectiveness.

(1) The *laboratory-based surveillance strategy* (strategy 1): this baseline strategy corresponds to the current surveillance programme for H7N9 in LBMs considered at high risk of H7N9 introduction in Vietnam. It involves the random sampling of 40 oropharyngeal swabs on chickens only, as they have been shown to be more susceptible to H7N9 infection than ducks [24]. Chickens are usually sampled twice a week at times between 8.00 and 10.00. All samples are then transported to an official diagnostic laboratory where they are all screened for the M gene using RT-PCR; those positive for the M gene are subsequently tested for the H7 gene using RT-PCR. As planned by the procedure, those positive for the H7 gene should be tested for the N9 gene. However, according to the World Animal Health Information System (WAHIS), H7 viruses have not yet been detected in Vietnam so testing for N9 was not included in the calculation of surveillance costs. On average, the delay between sampling and diagnostic test result communication is around 72 h.
(2) The *optimized laboratory-based surveillance strategy* (strategy 2): this first alternative strategy is similar to strategy 1 except that samples are collected at the time of the day that maximizes the probability of sampling at least one infectious bird, i.e. at the time of the highest within-LBM prevalence of infection, as predicted by the model.
(3) The *portable PCR surveillance strategy* (strategy 3): this second alternative strategy is similar to strategy 1 except that all samples are directly analysed on site for the H7 gene using the portable PCR device [25,26], assuming an average delay of 7 h between sampling and diagnostic test result communication.
(4) The *optimized portable PCR surveillance strategy* (strategy 4): this third alternative strategy is similar to strategy 3 except that samples are collected at the time of the day that maximizes the probability of sampling at least one infectious bird.

These four different surveillance strategies were coupled with three different sampling frequencies: once a week, twice a week and every day. The surveillance strategies were also assessed assuming that the LBM policy could change and forbid the presence of birds staying overnight, as this intervention strategy has been shown to be an effective control option for AIV [21,42].

#### 2.4.2. Comparison of the surveillance strategies

For a given combination of surveillance strategy, sampling frequency and overnight staying policy, the effectiveness was described by estimating the most likely day of detection post-introduction and the number of infected or infectious birds traded to farms or other LBMs at the most likely day of detection. The most likely day of detection was defined as the day when the cumulative probability of detection of at least one infectious bird became greater than 50% (electronic supplementary material, appendix). The weekly costs of H7N9 surveillance were also estimated for each strategy, with cost parameters provided by the SDAH (electronic supplementary material, appendix and table S1).

## 3. Results

### 3.1. Parameters for the LBM bird trading model

Table 2 presents parameter values related to the population dynamics of birds at Giếng Vuông LBM. This was a large LBM, with a high average daily number of birds (*n* = 7000). The predominant traded bird species reported was chicken (80%), with 25% of total number of birds staying overnight. The highest percentage of birds entering and leaving the LBM was reported to be at times between 2.00 and 6.00 and between 6.00 and 8.00, respectively.

**Table 2.** Parameter values related to population dynamics of birds at Giếng Vuông LBM, Lang Son province, northern Vietnam.

parameter	value
average daily number of birds entering the LBM	7000
percentage of birds present in the LBM	chickens: 80% ducks: 20%
percentage of birds staying overnight	25%
percentage of birds per type of destination	to trading places, including farms and other LBMs: 40% to slaughter places, including slaughter houses, restaurants and end-consumers: 60%
percentage of birds entering the LBM per time slot	2.00–3.00: 20% 3.00–4.00: 20% 4.00–5.00: 20% 5.00–6.00: 20% 6.00–7.00: 10% 7.00–8.00: 10%
percentage of birds leaving the LBM per time slot	4.00–5.00: 5% 5.00–6.00: 5% 6.00–7.00: 20% 7.00–8.00: 20% 8.00–9.00: 15% 9.00–10.00: 15% 10.00–11.00: 10% 11.00–12.00: 10%

### 3.2. Within-LBM H7N9 transmission model

**Figure 3.**
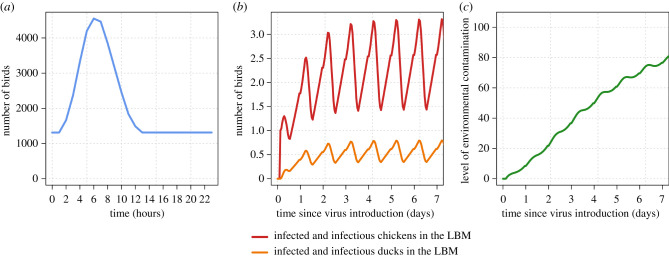
Population dynamics of birds (*a*), estimated number of infected and infectious birds (*b*) and level of environmental contamination (*c*) at Giếng Vuông LBM, Lang Son province, Vietnam.

Assuming an overnight stay of birds, the daily dynamic of infection was expected to reach a regular pattern around two to three days after virus introduction (figure 3). At night, when the overall population within the LBM is closed (no entry, no exit), the virus spread between the overnight-staying birds, resulting in the decrease in the number of susceptible birds and the increase in the number of infected or infectious birds. Given the model parameters, the virus was able to persist in the environment in the long term, likely due to the presence of birds staying overnight in the LBM allowing virus amplification. The level of environmental contamination was expected to reach a plateau after several days. When implementing a ban on the presence of birds staying overnight, the number of infected birds and the environmental contamination level remained very limited, resulting in the fade-out of the epidemic within the first few days (electronic supplementary material, appendix and figure S1). However, note that this intervention likely reduced the chance of virus detection before it disappears (see below).

### 3.3. Cost-effectiveness of surveillance strategies

**Figure 4.**
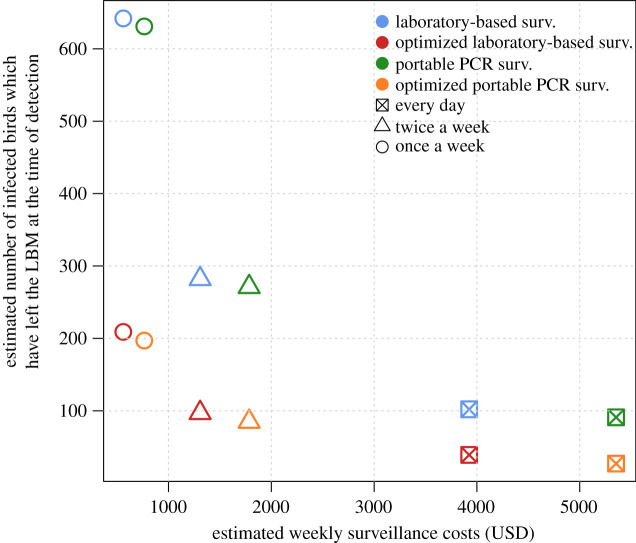
Estimated weekly costs (in USD) and estimated number of infected birds which have left the LBM at the day of detection for the different surveillance strategies at Giếng Vuông LBM, Lang Son province, Vietnam.

Based on model simulations, the time of the day when the prevalence was at its maximum, i.e. when the probability of sampling at least one infectious bird in the studied LBM was at its peak, was estimated at 1.00. This corresponds to the moment just before new susceptible birds are introduced into the LBM at the beginning of a working day. In figure 4, the laboratory-based surveillance strategy involving the sampling of chickens twice a week (blue triangle) corresponds to the baseline strategy for the surveillance of H7N9 in LBMs in Vietnam. Using its equivalent optimized strategy (red triangle) decreased the number of infected birds by approximately 60% for the same weekly surveillance cost (1308 USD) (electronic supplementary material, appendix and table S2). Using the optimized laboratory-based surveillance strategy once a week (red circle) decreased both the number of birds and the costs by approximately 30% and 60%, respectively. Similar results were obtained when comparing the portable PCR surveillance strategy (green) and its equivalent optimized strategy (orange). Also, when compared with the optimized laboratory-based surveillance strategy (red), using the optimized portable PCR surveillance strategy (orange) decreased the number of infected birds by approximately 10–30% (depending on the sampling frequency) but increased the weekly surveillance costs by approximately 30%.

The sensitivity analysis (electronic supplementary material, appendix and figures S2, S3 and S4) showed that changes in the three parameter values for which no information was available in the literature (*w*, *Q* and *ξ*) did not impact the time of the day that maximized the probability of sampling at least one infectious bird (i.e. 1.00) and the effectiveness of the surveillance strategies.

## 4. Discussion

Model outputs suggested that H7N9 transmission could be sustained for weeks following the introduction of a single infectious bird through virus amplification and transmission within the LBM among birds staying overnight, which is consistent with results from a previous study on H5N1 [42]. The LBM system thus acted as a reservoir of infection for newly introduced susceptible birds. The model predicted that, using the current laboratory-based surveillance strategy for H7N9 in LBMs, several hundreds of infected birds would have already been sold to farms or other LBMs by the time the virus is expected to be detected in the LBM. A portable PCR method to detect H7N9 virus at the LBM with similar sensitivity (98%) and specificity (100%) to laboratory-based PCR assays was recently introduced with the aim of reducing the time delay between sampling and diagnostic test result communication [26]. Model outputs showed that using this alternative surveillance strategy twice a week would lead to similar surveillance costs and to only a slightly improved effectiveness as compared to the current laboratory-based surveillance strategy.

Model outputs also indicated that both laboratory-based and portable PCR surveillance strategies could be further optimized by sampling the birds staying at night just before new susceptible birds are introduced at the beginning of an opening LBM day. However, this would involve sampling and testing birds very early in the morning, which should be discussed with local communities and stakeholders as it may not be feasible in certain settings. Another limitation to consider is that scaling up surveillance systems involving a portable PCR device could be challenging due to constraints on the maximum number of samples that can be tested at once, while economies of scale can be achieved under laboratory-based strategies.

Birds infected with H7N9 generally show mild clinical signs but they can excrete the virus for 5 to 8 days [24]. Traders reported keeping birds for a few days within the LBM until being sold, housing them in cages overnight. These birds staying for longer time at LBMs are more likely to get infected and play an important role in the maintenance and amplification of H7N9 within the LBM. Model outputs showed that banning birds overnight at LBM resulted in the fade-out of the epidemic within the first few days but reduced the chance of virus detection before fade-out. Importantly, this intervention strategy could represent a less disruptive, more sustainable and effective measure than LBM closure, especially since the latter approach requires the virus to be detected what can take weeks (figure 4). Furthermore, LBM closure has been associated with substantial costs for the poultry industry and the emergence of illegal trade in some LBMs or neighbourhoods, making disease monitoring and control more difficult [43–45].

Given the diversity of poultry species present in LBMs, one should be cautious when attempting to generalize these results as variation in H7N9 transmission may be caused by different levels of susceptibility to H7N9 infection. Indeed, susceptibility to H7N9 has been reported to vary between species, with chickens being generally considered to be more susceptible than ducks [27,30]. Moreover, spent hens have been identified as one of the main poultry categories imported from China to Vietnam for consumption [46]. Limited information was available on the different species present within the LBMs, preventing more information to be integrated into the model. The model formulation assumed homogeneous mixing, meaning that birds could uniformly and randomly contact each other in the LBM, which was considered as an acceptable assumption given the high number of bird stalls, the limited physical separations and distances between stalls, the frequent handling of birds by sellers and buyers in LBMs. Another limitation of the model was the limited information regarding H7N9 virus survival in the environment. Environmental contamination by H7N9 in LBMs has been documented with positive samples retrieved in faeces, cages and floor, but also in de-feathering machines and chopping tools [10]. However, little is known on the virus inactivation rate across time for various environmental factors, such as temperature and humidity. While no information was available on the number of excreted infectious doses and on the contact rate with one infectious dose, the sensitivity analysis showed that the assumptions related to these parameters did not impact the conclusions of this study. Finally, note that model outputs showed that the virus was able to persist in the environment in the long term, suggesting that the sensitivity of PCR surveillance strategies based on environmental sampling needs to be further investigated.

The Giếng Vuông LBM is one of the 17 LBMs in northern Vietnam that had been identified as at high risk of becoming H7N9 infected due to potential cross-border trade of birds from China. This is a unique LBM with its proper structure and functioning. Thus, its bird population dynamics differ from those of other LBMs, resulting in different optimized times of sampling. Moreover, its risk of becoming H7N9 infected has significantly decreased following the implementation of a vaccination campaign for H7N9 in China in 2017 [47]. Thus, to allow stakeholders to apply this analytical framework to other LBM systems and AIV, a Web application was developed using the shiny package [48] and made publicly available with a graphical user interface in html format at: https://envt-inra.shinyapps.io/optimia/. The Web application allowed model simulation outputs to be adapted to other LBMs identified by Vietnamese veterinary services as at high risk of H7N9 introduction but which were not described in this paper for clarity reasons. Such Web application applied in LBMs could help in reducing zoonotic and pandemic risks posed by similar emerging AIV.

## Data accessibility

A Web application was developed using the shiny package and made publicly available with a graphical user interface in html format at: https://envt-inra.shinyapps.io/optimia/. The Web application allowed to provide model simulation outputs adapted to other LBMs identified by Vietnamese veterinary services as at high risk of H7N9 introduction but which were not described in this paper for clarity reasons. The data are provided in the electronic supplementary material.
